# Metamorphosis and lncRNAs: A Close Relationship

**DOI:** 10.1002/dvg.70052

**Published:** 2026-04-13

**Authors:** H. Herrera‐Orozco, H. A. Pérez‐Mendoza

**Affiliations:** ^1^ Posgrado en Ciencias Biológicas, UNAM Unidad de Posgrado Edificio D primer pisoCiudad Universitaria, CDMX México Mexico; ^2^ Laboratorio de Ecología Evolutiva y Conservación de Anfibios y Reptiles, Facultad de Estudios Superiores Iztacala UNAM Estado de México Mexico

**Keywords:** amphibian, evolutionary conservation, functional annotation, lncRNAs, metamorphosis

## Abstract

The classical definition of metamorphosis is a post‐embryonic transformation, such as from a tadpole to a froglet. However, recent studies suggest this process occurs to some degree in all vertebrates, as the underlying endocrine and molecular pathways are highly conserved. With the advent of high‐throughput sequencing, transcriptomic data for non‐model species has revealed that protein‐coding genes represent only a small fraction of the genome. In contrast, most transcriptional output produces non‐coding RNAs with vital regulatory functions. Among these, long non‐coding RNAs are a diverse and important class known to regulate gene expression at multiple levels and across various biological contexts. Despite their established importance, the study of lncRNAs across the tree of life remains an open field, crucial for understanding their potential roles and evolutionary conservation. This work summarizes the roles of lncRNAs as regulatory molecules, their functions in development and metamorphosis, computational strategies for their characterization, and the challenges and opportunities of studying them in non‐model species.

## 1 Introduction

Metamorphosis (Greek *meta‐* “change” and ‐*morphos* “form”) describes the ontogenic shift in life‐cycle stage from a larva into a juvenile, which is almost always accompanied by major morphological changes and habitat relocation that allow the exploitation of distinct niches (Bishop et al. 2006). From the basal lineage (lampreys) metamorphosis occurs across vertebrates. Teleost fishes exhibit diverse metamorphic strategies ranging from subtle physiological adjustments to complete ecological transitions (McMenamin and Parichy 2013). While other vertebrate groups demonstrate more attenuated forms of metamorphosis, these processes share a conserved regulatory framework of molecular signaling pathways (Paris and Laudet 2008). Amphibians remain the most iconic exemplars of this process, with their dramatic transformation having served as a model system for developmental biologists for over a century.

Amphibian metamorphosis is an endocrine‐driven process where thyroid hormone (TH), and corticosteroids levels direct morphological, physiological, and biochemical changes, including tail reabsorption (Wang et al. 2019), hind limb development (Tanizaki et al. 2022), and intestine remodeling (Tanizaki, Zhang, et al. 2023). During metamorphosis, TH reaches peripheral tissues and enters the nucleus binding to its receptor, recruiting specific Transcription Factors (TF) eliciting transcription of metamorphosis‐related genes (Shibata et al. 2020).

TH binding sites occur within intergenic regions (Raj et al. 2023), suggesting that many metamorphosis‐associated genes are transcribed from non‐protein‐coding loci. This observation aligns with the well‐established fact that while only about 2% of the genome encodes proteins, ~80% is actively transcribed, producing a diverse array of non‐coding RNAs (ncRNAs) with specialized functions (Poliseno et al. 2024).

NcRNAs are transcripts that have little or no coding potential but have structural or regulatory functions across various cellular contexts (Ali et al. 2021). NcRNAs are further sub‐classified by size into small ncRNAs (< 200 nucleotides, miRNAs, piRNAs, snRNAs); and long ncRNAs (lncRNAs) which exceed 200 nucleotides in length (Chen and Kim 2024). This definition is arbitrary because it was proposed as a clearcut guide to separate lncRNAs as regulatory transcripts from sncRNAs that perform structural roles. A more refined definition of lncRNAs is transcripts over 500 nucleotides with little or no coding potential, transcribed by RNA polymerase II (Pol II), 5′capped, 3′ polyadenylated, and even spliced (Mattick et al. 2023). LncRNAs represent an additional layer of genetic regulation as distinct transcripts are correlated with complex biological phenomena (Statello et al. 2021) such as cell proliferation (Li, Ran, and Qiao 2023), differentiation (Wu, Dai, et al. 2023), DNA damage response (Luo et al. 2024), and senescence (Qin et al. 2024).

We aim to synthesize the knowledge of lncRNA regulation of metamorphosis as part of vertebrate ontogeny the extent of their evolutionary conservation, bioinformatic approaches, and current challenges and opportunities to their discovery and annotation.

## 2 LncRNA Transcription

### 2.1 Pervasive Transcription

An important concept to consider when discussing lncRNA biogenesis is pervasive transcription, which refers to the widespread production of RNA molecules from genomic regions outside protein‐coding genes (PCGs) and classical structural or regulatory loci (e.g., tRNAs, rRNAs, snRNAs) in eukaryotic genomes. The term pervasive does not necessarily imply a lack of function—many of these transcripts remain uncharacterized—but rather emphasizes the extensive, genome‐wide nature of this phenomenon (Jensen et al. 2013).

In eukaryotes, RNA polymerase II (Pol II) is considered a major driver of pervasive transcription, as it can initiate transcription from a broad range of genomic sites, particularly nucleosome‐free regions (De Dieuleveult et al. 2016; Ozonov and van Nimwegen 2013). Most Pol II promoters exhibit bidirectional activity: while most Pol II complexes initiate transcription in the canonical 5′–3′ direction, a fraction initiate antisense transcription from the same promoter region (Ahmad et al. 2021). This bidirectional transcription gives rise to a diverse repertoire of non‐coding RNAs, including enhancer RNAs (eRNAs), promoter upstream transcripts (PROMPTs), and other long intergenic non‐coding RNAs (lincRNAs) (Napoli et al. 2022; Yang et al. 2024), as well as numerous unstable cryptic transcripts that are rapidly degraded by the nuclear exosome and other RNA surveillance pathways (Berretta and Morillon 2009).

The evolutionary advantage of pervasive transcription has been questioned, as dysregulation of this process may be highly detrimental to the cell. Potential consequences of loose transcriptional control include transcriptional interference with PCGs, genome instability, and titration of limiting cellular factors (Villa and Porrua 2023). However, although random transcriptional interference may be harmful, several well‐documented cases demonstrate that transcriptional interference can function as a regulatory mechanism in specific biological contexts, such as quiescence (Nevers et al. 2018), homeostasis—through Zap1 downregulation—(Haidara et al. 2022), and metabolite control (Shuman 2020). It is important to note that many of these conclusions are derived from studies in budding yeast, and their scalability to metazoans remains to be fully determined.

Although a comprehensive discussion of the biological impact of pervasive transcription is beyond the scope of this work, it is reasonable to view it as a “controlled risk” for the cell (Villa and Porrua 2023). Accordingly, a sensible null hypothesis when studying pervasively transcribed non‐coding RNAs is that most transcripts have minimal impact on reproductive fitness (Ponting and Haerty 2022).

### 2.2 LncRNA Transcription Is Different From mRNAs

To understand lncRNA in gene regulation first we need to understand that lncRNA transcription is regulated differently than mRNAs. In eukaryotic cells Pol II is in charge of producing all pre‐mRNAs from PCGs which are co‐transcriptionally matured by 5′‐capping, splicing, and 3′‐polyadenilation (Schier and Taatjes 2020). Pre‐mRNA transcription is a well‐defined process determined by the 5′‐cap and 3′‐polyadenylation sites marking the Transcription Start Sites (TSS) and Transcription End Sites (TES) respectively.

Although lncRNAs are also transcribed by Pol II, their transcriptional regulation, initiation, termination and processing are less tightly constrained than those of mRNAs. In this section, we aim to delineate the main differences between lncRNA and mRNA transcription and processing.

#### 2.2.1 Transcription Initiation

Although Pol II is also responsible for the synthesis of lncRNAs, lncRNA transcription is still poorly understood, as it is often tissue‐specific and occurs at low levels (Nojima and Proudfoot 2022). Analysis of the core promoter regions of experimentally validated human and lncRNA *loci* (−50 to +30 bp relative to the TSS) revealed that their three‐dimensional promoter structure resembles those of PCGs particularly in two regions: an Initiator Element (INR) hexanucleotide around the TSS and a TATA box (octa nucleotide around −28 bp). However, these elements differ in terms of their frequency and sequence characteristics; notably, TATA box occurrence is significantly rare in lncRNA promoters, and the informational entropy at each position is higher compared to the PCG core promoter leading to weaker and less constrained motifs for lncRNAs that are crucial to transcriptional regulation of PCGs (Savina et al. 2023).

Transcription initiation at PCG promoters requires pioneer TFs that recruit chromatin‐remodeling complexes, facilitating the transition of chromatin into an accessible state and enabling subsequent binding of specific downstream TFs that drive transcription initiation (Schier and Taatjes 2020). This level of regulatory complexity is not universally shared by lncRNA promoters. Instead, lncRNA transcription frequently occurs within pre‐existing accessible chromatin regions, effectively “hitchhiking” on active transcriptional hubs and often correlating with the expression of nearby PCGs. This phenomenon is exemplified by bidirectionally transcribed enhancer RNAs (eRNAs) and antisense lncRNAs (Nojima and Proudfoot 2022).

Moreover, lncRNAs can contribute to the maintenance of open chromatin states through the formation of R‐loops—RNA–DNA hybrid structures formed when the RNA transcript anneals to one strand of the DNA duplex. R‐loops are particularly enriched at Pol II promoters and are strongly associated with antisense lncRNA transcription (Tan‐Wong et al. 2019), supporting the notion that chromatin architecture and RNA structures, rather than strict sequence motifs may be acting as key regulatory features for lncRNA transcription (Nojima and Proudfoot 2022).

#### 2.2.2 Transcription Termination

Transcription termination of PCGs can occur through two non‐mutually exclusive mechanisms: the allosteric model and the torpedo model. In the allosteric model, transcription of the polyadenylation site induces conformational changes in Pol II, including dephosphorylation of the elongation factor SPT5, which slows transcription and facilitates termination. This process enables the exonuclease XRN2 to degrade the downstream RNA and promote Pol II release from the DNA template (Rodríguez‐Molina et al. 2023). In the torpedo model, XRN2 is recruited to the exposed 5′ end of the cleaved pre‐mRNA and degrades the RNA in a processive manner until it catches up with Pol II, triggering polymerase disengagement (Yanagisawa et al. 2024).

Transcription termination of lncRNAs involves additional specialized protein complexes, most notably Integrator, Restrictor, and Microprocessor (Nojima and Proudfoot 2022). The Integrator complex associates with paused or slowed Pol II and mediates cleavage of the nascent RNA, a mechanism initially characterized for snRNAs but now recognized to act on subsets of lncRNAs (Barra et al. 2020; Razew et al. 2024). Alternatively, the Restrictor complex terminates transcription independently of RNA cleavage; instead, its components PNUTS and WDR82 promote dephosphorylation of SPT5, leading to Pol II release (Russo et al. 2023). In addition, some lncRNAs are processed by the Microprocessor complex, in which co‐transcriptional cleavage of embedded pre‐miRNAs within lincRNAs induces downstream Pol II termination independently of the canonical polyadenylation‐dependent pathway (Dhir et al. 2015).

A few lncRNAs (particularly PROMPTs and eRNAs) are under strong genomic surveillance by the NNS termination complex—Nrd1‐Nab3‐Sen1—(Rodríguez‐Molina et al. 2023). In this pathway, Nrd1 and Nab3 are recruited to nascent transcripts through sequence‐specific motifs and subsequently engage the helicase Sen1, which promotes transcript release. The released RNA is then rapidly degraded by the nuclear exosome (Xie et al. 2023). This mechanism is generally interpreted as a transcription attenuation system and a fail‐safe to prevent readthrough transcription into downstream genes (Nojima and Proudfoot 2022). How certain lncRNAs evade NNS‐mediated termination and achieve stable expression remains an open question and an important avenue for future research.

### 2.3 Alternative Transcription and Isoform‐Complexity

Transcription is a complex biological process regulated at multiple levels. Through the interaction of diverse molecular players, a single genomic locus can give rise to multiple RNA products, providing cells with the ability to adapt to different conditions without requiring additional genetic material (Bone and Inman 2025), the different RNA products generated from the same locus are named isoforms.

#### 2.3.1 Alternative Transcription Start Sites

Promoters can have a flexible TSS were depending on epigenetic modifications, cellular context (e.g., hypoxia), or developmental stage differential isoform selection may be enacted on the locus regulating gene expression in a tissue or context specific manner (Alfonso‐Gonzalez and Hilgers 2024). For example, Xist (master regulator of X chromosome inactivation) possesses 3 putative promoters termed P0, P1 and P2. P1 is the minimal promoter region located right upstream of the major TSS, P0 is located 6.5Kb upstream of the TSS and produces unstable Xist transcripts; alternatively, P2 is located around 1.5Kb downstream of the TSS (inside exon 1) and produces a stable transcript (Luchsinger‐Morcelle et al. 2024). Recent data suggest that P2 could be acting as an enhancer instead of a promoter (Samanta et al. 2022), questioning the relevance of alternative transcription as a regulatory mechanism.

Some authors suggest that most alternative transcriptional initiation events may not be adaptive, but instead represent molecular noise or error, in which one TSS is optimal and associated with proper gene function, whereas alternative sites arise from imprecise transcription initiation (Xu et al. 2019). Whether alternative transcription initiation reflects complex gene regulatory mechanisms or molecular errors remains an open question, as the adaptive value of such underlying molecular variation may lie in its potential to provide raw material upon which natural selection can act.

#### 2.3.2 Alternative Polyadenylation and 3′‐Processing

Another major contributor to transcriptional and post‐transcriptional complexity in lncRNAs is alternative 3′ polyadenylation (APA) and cleavage (Ziegler and Kretz 2017). A substantial proportion of lncRNAs (~70%) exhibit APA patterns that differ from those observed in mRNAs. In lncRNAs, polyadenylation sites are frequently located upstream of the most 3′ exon, whereas in mRNAs these sites are typically found within the terminal exon (Xu et al. 2019). The most common APA outcome in lncRNAs is the generation of transcript isoforms with variable 3′ UTR lengths (Xu et al. 2022), a feature that can influence subcellular localization, secondary structure, and functional interactions of the RNA molecule (Bone and Inman 2025).

The lncRNA NEAT1 exemplifies the functional consequences of alternative 3′ end processing, as it produces two major isoforms: NEAT1v1 and NEAT1v2, which differ primarily in their 3′ end processing. NEAT1v1 (~3.7 kb) is polyadenylated, whereas NEAT1v2 is non‐polyadenylated and substantially longer (~23 kb) (Xu et al. 2022). Both isoforms are required for paraspeckle formation: NEAT1v2 acts as the structural scaffold by first associating with the RNA‐binding proteins p54nrb (NONO) and SFPQ, subsequently recruiting NEAT1v1 and PSPC1 to stabilize the paraspeckle structure (Wang et al. 2020). Disruption of this regulatory balance, such as overexpression of NEAT1v1, leads to reduced paraspeckle assembly and has been implicated in the progression of neurodegenerative diseases (Briata and Gherzi 2020).

Some lncRNAs bypass the canonical polyadenylation pathway altogether and instead undergo alternative 3′ end processing mediated by RNase P cleavage (Khan et al. 2021). RNase P–dependent processing generates a highly stable U•A–U major‐groove RNA triplex structure through cleavage of an 11‐nt A‐rich tract from its complementary downstream 3′ U‐rich sequence. This configuration allows the A‐rich tract to interact with an upstream U‐rich internal loop, as exemplified by the lncRNA MALAT1 (Skeparnias et al. 2024). Recognition by RNase P appears to depend on the formation of a specific structural motif at the 3′ end of the nascent lncRNA that partially mimics the tRNA “elbow,” thereby facilitating RNase P recruitment, a mechanism also described for NEAT1 (Skeparnias and Zhang 2024).

#### 2.3.3 Alternative Splicing

Similarly to mRNAs, lncRNAs are composed of introns and exons and contain regulatory elements at their 5′ and 3′ ends, making them susceptible to splicing. However, compared with PCGs, lncRNAs exhibit lower splicing efficiency, as reflected by the smaller number of transcript isoforms produced per gene. In humans, approximately 16,000 lncRNA genes give rise to ~28,000 transcripts, resulting in a substantially lower transcript‐to‐gene ratio than that observed for PCGs (Khan et al. 2021). This reduced splicing efficiency may arise from intrinsic structural differences between lncRNAs and PCGs, including a lower average number of exons, differences in intron length, divergence in 5′ and 3′ splice‐site consensus sequences at branch points and donor/acceptor sites, and interactions with distinct or specialized splicing factors (Abou Alezz et al. 2020).

Nonetheless, splicing in lncRNAs has emerged as an important regulatory mechanism. For example, alternative splicing of the lncRNA SNHG19 promotes chemoresistance in hepatoblastoma by downregulating snoRNA expression (Zhen et al. 2023). Examples of this can be found in other biological contexts: in rice, alternative splicing of the lncRNA LAIR generates multiple isoforms that fine‐tune expression of the LRK1 locus through differential binding to the epigenetic regulators OsMOF and OsWDR5, which control H4K16ac and H3K5me3 histone marks, respectively, thereby modulating yield‐related traits (Wang et al. 2024). Similarly, distinct isoforms of lncFAM200B downregulate Cyclin D1 mRNA, inhibiting preadipocyte proliferation during bovine adipogenesis and correlating with body measurement traits in cattle. Evidence of lncRNA splice variants highlights the relevance of these molecules on different aspects of gene expression regulation and cell biology.

Collectively, these observations underscore that lncRNA transcription operates within a complex, context‐dependent regulatory landscape. Despite growing mechanistic insight, many fundamental questions regarding the regulation, processing, and functional relevance of lncRNAs remain open and will require targeted experimental validation.

## 3 LncRNAs In Gene Regulation

LncRNAs regulate gene expression from chromatin structural organization to the control of transcription, translation, RNA localization, and stability. Their functions arise from their ability to interact with RNAs (Zheng, Gao, et al. 2023), proteins (Li, Sun, et al. 2021), and DNA (Lee et al. 2024). Subcellular localization of lncRNAs is linked to their function; a significant proportion of lncRNAs is retained in the nucleus, where they associate with chromatin to regulate gene expression (Tong and Yin 2021). Cytoplasmic lncRNAs function as molecular decoys, modulating the activity of other RNAs or proteins, and their stability and turnover (Noh et al. 2018).

### 3.1 Nuclear LncRNAs

**FIGURE 1:**
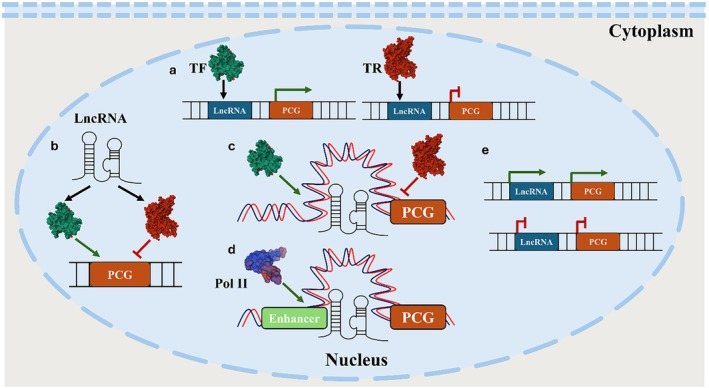
Gene regulation by lncRNAs inside the nucleus. LncRNAs regulate gene expression within the nucleus through multiple mechanisms: (a) lncRNA loci can recruit transcriptional activators or repressors, functioning as enhancers or insulators for nearby protein‐coding genes (PCGs); (b) lncRNA transcripts can recruit TFs to PCG loci; (c) lncRNAs can recruit chromatin‐remodeling complexes to PCGs; (d) lncRNAs can modulate chromatin architecture, thereby regulating accessibility to PCG loci; and (e) transcription of the lncRNA itself can influence PCG transcription by generating a permissive chromatin environment (e.g., a transcriptional bubble) that increases accessibility of nearby PCGs, whereas silencing of the lncRNA locus can also result in silencing of the associated PCG.

LncRNAs regulate chromatin architecture on the same chromosome from which they are transcribed (*in cis*), or targeting loci present in different chromosomes (*in trans*) (Mishra and Kanduri 2019). Their influence on chromatin can be direct, through physical interactions with DNA and recruitment of TFs, RNA‐binding proteins (RBPs), or histone‐modifying enzymes; or indirect, acting as scaffolds that stabilize chromatin‐remodeling complexes without directly binding DNA (Tang et al. 2023). Up to 60% of nuclear lncRNAs are found associated with transcriptionally active chromatin regions near their own transcriptional sites (Werner and Ruthenburg 2015), supporting their role as fine tuners of local gene expression (Bell et al. 2018; Bonetti et al. 2020). Nuclear roles of lncRNAs are shown in Figure 1.

#### 3.1.1 Activity in *Cis*

A classic example of cis‐acting chromatin regulation is X chromosome inactivation in mammals, mediated by the lncRNA Xist (Brockdorff et al. 2020). Xist is randomly expressed from one of the two X chromosomes; it spreads along the entire chromosome, recruiting transcriptional repressors and chromatin modifiers to establish the X‐inactivation center (Tian et al. 2010), ultimately leading to chromosome‐wide silencing through heterochromatin formation (Wang, Min, et al. 2021).

LncRNAs influence spatial accessibility of genomic regions through interactions with TFs. Kancr enhances Kdm2b transcription by binding hnRNPAB, facilitating chromatin looping between the enhancer and promoter (Li et al. 2020). These effects can also extend beyond local interactions; HOTAIRM1 tethers to its own locus to reorganize the 3D chromatin structure around the HOXA cluster (~120 kb) sustaining its transcription (Shi et al. 2020).

#### 3.1.2 Trans‐Acting lncRNAs

LncRNAs influence gene expression across the genome (*in trans*). A key example involves CCCTC‐binding factor (CTCF), a conserved regulator of 3D chromatin architecture (Dehingia et al. 2022). Around 5000 human lncRNAs are predicted to bind CTCF through RNA‐specific motifs (Kuang and Wang 2020), suggesting a potentially widespread mechanism—though these interactions require further validation. LncRNA Jpx (Tian et al. 2010), performs genome‐wide regulation by displacing CTCF from low‐affinity sites, reshaping chromatin looping (Oh et al. 2021). Beyond these broad effects, lncRNA–CTCF regulation can also occur locally; lncRNA PACERR forms a complex with CTCF and recruits the histone acetyltransferase p300, enhancing histone acetylation thus enabling neighboring gene activation (Liu, Wang, et al. 2022).

#### 3.1.3 Epigenetic Regulation

LncRNAs influence chromatin organization by guiding histone‐modifying complexes to precise genomic locations, such as the Polycomb Repressive Complexes (PRCs). Polycomb proteins are evolutionarily conserved transcriptional repression complexes (PRC1 and PRC2) (Blackledge and Klose 2021; Piunti and Shilatifard 2021). LncRNAs have been found to direct PRCs to their target sites, establishing localized chromatin repression (Chi et al. 2017; Kim et al. 2017). AIRN acts as a master regulator of localized gene silencing across a 15 Mb domain by binding to multiple regulatory DNA elements and facilitating the recruitment of both PRC1 and PRC2 (Braceros et al. 2023). This model suggests that similar lncRNA‐mediated mechanisms may govern repression in other chromosomal contexts.

#### 3.1.4 DNA–LncRNA Interactions

LncRNAs orchestrate gene expression and shape chromatin architecture through direct interactions with DNA across the entire genome. LncRNAs form R‐loops and triplex structures that not only regulate specific genes but also help organize higher‐order features like topologically associating domains (TADs) and nuclear compartments (Soibam and Zhamangaraeva 2021), positioning lncRNAs as key regulators of genome architecture.

LncRNAs direct TFs via sequence‐specific hybridization, exemplified by APOLO, which forms R‐loops (Niehrs and Luke 2020) to recruit LHP1, a component of PRC1 (Ariel et al. 2020). R‐loop‐mediated regulation is critical in eukaryotes, as demonstrated by TERRA, which mediates RAD51‐dependent R‐loop formation to maintain telomere length (Lalonde and Chartrand 2020). Alternatively, lncRNAs engage DNA through Hoogsteen‐bonded triplex helices in the major groove (Leisegang et al. 2024), guided by stablished sequence characteristics (purine bias, nucleosome proximity and positively charged H3 N‐tails) (Greifenstein et al. 2021). These principles allow for nuanced regulatory outcomes: some lncRNAs repress transcription by recruiting silencing complexes, like LINC00525 via EZH2 (Fang et al. 2021); while others, like RAINY, activate gene expression (Westemeier‐Rice et al. 2025).

#### 3.1.5 Transcript‐Independent Regulation

Some lncRNAs regulate neighboring genes through DNA elements within their locus or through the act of transcription itself, rather than through the function of the RNA molecule. A well‐characterized example is the lncRNA BENDR, whose promoter functions as a cis‐regulatory element controlling expression of the adjacent downstream gene Bend4. Deletion of 750 bp of the BENDR promoter reduced Bend4 expression by ~60%, indicating that this region contains enhancer elements required for proper activation of Bend4. However, insertion of a premature polyadenylation signal in the first BENDR exon abolished transcription of the mature BENDR transcript yet had no effect on Bend4 expression. These results demonstrate that the BENDR is not required for Bend4 activation. Instead, the regulatory effect is mediated by DNA cis‐regulatory elements embedded within the promoter region of the BENDR locus (Engreitz et al. 2016).

Similarly, the promoter of the lncRNA HNF1A‐AS1, also termed HASTER, functions as a regulatory element controlling expression of the adjacent HNF1A gene. The HASTER promoter initially activates HNF1A transcription, which in turn promotes HNF1A‐AS1 expression. Subsequently, the HNF1A protein binds to the HASTER promoter, disrupting enhancer–promoter interactions and establishing a negative feedback loop that restricts HNF1A expression (Beucher et al. 2022).

These cases underscore dual lncRNA function: the DNA element itself can act as a regulatory hub, while the lncRNA transcript may carry out separate roles. Experimental strategies should aim to dissect the respective contributions of the genomic locus, the act of transcription, and the RNA product.

#### 3.1.6 Enhancer‐Associated lncRNAs

Enhancer‐associated lncRNAs fall into two main categories: enhancer RNAs (eRNAs), which are bidirectionally transcribed, unspliced, poly‐A‐deficient transcripts rapidly degraded by the nuclear exosome (Yin and Shen 2023); and enhancer‐associated long intergenic ncRNAs (elincRNAs), which are more stable, unidirectionally transcribed, spliced, and marked by distinct chromatin signatures (H3K4me1 and H3K27ac) (Kang et al. 2021).

Enhancers are architectural anchors mediating chromatin looping between distal regulatory elements and promoters. It is known that eRNAs actively modulate this process, guiding histone modifying complexes (Cuomo et al. 2025), binding specific TFs (Yuan et al. 2024), and even forming R‐loops that influence chromatin dynamics (Akiki et al. 2024). ERNA expression levels correlate with enhancer activity (Kristjánsdóttir et al. 2020), and evidence indicates that elincRNA splicing plays an active role in enhancer‐mediated regulation (Tan and Marques 2022). Despite their functional similarities, enhancer‐derived RNAs exhibit distinct molecular characteristics (Betti et al. 2025). Current literature often uses the terms eRNAs and elincRNAs interchangeably, obscuring whether biochemical differences in stability and processing reflect distinct functional classes or represent a continuum of enhancer‐associated transcripts.

Significant studies come from super‐enhancers (SEs): large (10–60 kb) regulatory hubs comprising clustered enhancer elements, abundant TFs, Mediator Complex, and characteristic histone marks (Ma et al. 2025). SE‐derived lncRNAs drive expression of multiple genes correlated among each other and with complex phenotypes by modulating three‐dimensional chromatin architecture in long‐range interaction underscoring their importance in genome organization (Lavaud et al. 2024).

### 3.2 Cytoplasmic lncRNAs

**FIGURE 2:**
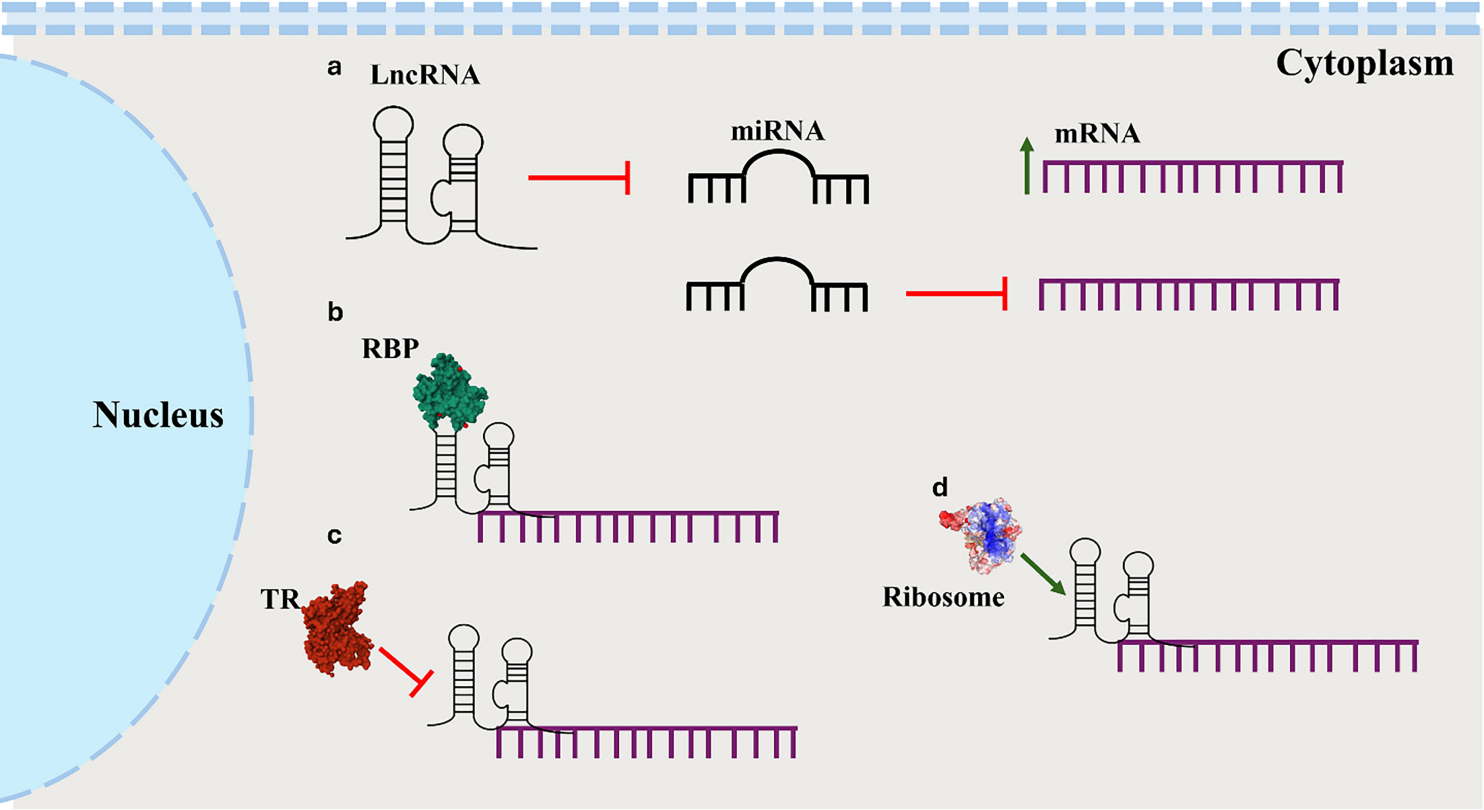
Gene regulation by lncRNAs in the cytoplasm. In the cytoplasm, lncRNAs exert post‐transcriptional regulation of gene expression through several mechanisms: (a) acting as competing endogenous RNAs (ceRNAs) by sequestering microRNAs; (b) regulating mRNA stability by recruiting RBPs to either protect transcripts from degradation or promote their decay; (c) inhibiting translation by recruiting translational repressors or directly interfering with ribosome assembly on target mRNAs; and (d) enhancing translation by facilitating ribosome recruitment, thereby increasing translational efficiency.

LncRNAs are exported into the cytosol in a 5′‐first manner through nuclear pores (Ashkenazy‐Titelman et al. 2022). This export mechanism is facilitated by their mRNA‐like structural features (Khan et al. 2023). In the cytosol lncRNAs undergo specific but still poorly understood sorting mechanisms that delivers them to distinct sub‐cellular compartments (Statello et al. 2021), where they act as modulators of mRNA stability, translational machinery regulators, molecular decoys, or components of signaling pathways (Figure 2).

#### 3.2.1 mRNA Stability and Translational Modulation

LncRNAs exert post‐transcriptional processing through the Staufen1 (STAU1)‐mediated mRNA decay pathway. STAU1 is an RBP that recognizes lncRNA–mRNA duplexes, where each RNA contributes one half of the STAU1‐binding domain—known as half‐STAU1‐binding site RNAs (½‐sbs RNAs)—typically formed by Alu repeats (Gong and Maquat 2011). In the duplex, STAU1 binds and recruits UPF2, leading to UPF1 helicase activity and mRNA degradation (Gowravaram et al. 2019). This mechanism has been exemplified by TINCR, which modulates tissue differentiation, proliferation and apoptosis by binding to multiple mRNAs (Xu et al. 2015).

LncRNAs enhance gene expression by stabilizing mRNAs, often through interactions with RBPs. For instance, FAM83‐AS1 recruits Fibrillarin to the FAM83 mRNA, shielding it from degradation (Wang, Zhao, et al. 2021), while SNHG16 facilitates the binding of EIF4A3 to RhoU mRNA, enhancing its stability (Ren et al. 2022). Additionally, lncRNAs can prevent miRNA‐mediated degradation by blocking miRNA response elements (MREs) on target mRNAs. An example is GSTu1‐AS1, which forms a duplex with its corresponding mRNA, protecting it from degradation by miR‐8525‐5p (Zhu et al. 2021).

LncRNAs modulate gene expression at the translational level. For instance, lincRNA‐p21 represses β‐catenin and JUNB translation by binding to multiple regions of their transcripts—including both the 5′ and 3′ UTR. LincRNA‐p21 forms a complex that recruits the translational repressor RCK, leading to ribosome drop‐off and inhibition of protein synthesis (Yoon et al. 2012), a similar role was reported for LncMyoD during skeletal Muscle Differentiation (Gong et al. 2015).

#### 3.2.2 Competitive Endogenous RNAs

Competitive Endogenous RNAs (ceRNAs) are lncRNAs that possess multiple MREs, identical to those present in mRNAs (Li, Xu, and Gao 2023). CeRNAs use this MREs to sequester (or “sponge”) miRNAs allowing the active translation of the original mRNA target (Asadi et al. 2023). This is one of the most studied regulatory mechanisms exerted by lncRNAs in multiple biological processes—mostly health related issues—such as: different types of cancer (Li, Yao, and Wang 2023; Wu, Zhong, et al. 2023), neurodegenerative diseases (Asadi et al. 2023) and autoimmune diseases (Zhang, Yang, et al. 2023). This mechanism allows for a bidirectional regulatory network shared between the coding and non‐coding transcriptome through a common target, making it a solid foundation for integrative expression analysis.

## 4 LncRNAs in Development and Metamorphosis

Vertebrates share a developmental plan that starts with the fusion of two mature germ cells forming a totipotent zygote that differentiates into pluripotent stem cells (PSCs) that will form the entire organism. A small subset of evolutionarily‐conserved lncRNAs plays essential roles during early embryogenesis, while species‐specific lncRNAs tend to emerge in later developmental stages (Sarropoulos et al. 2019). LncRNAs exhibit higher tissue specificity than mRNAs across tissues, reinforcing their role as key regulators of cell identity and fate (Mattioli et al. 2019). LncRNAs act as mediators of pluripotency, lineage specification, and organogenesis, positioning them as central players in developmental biology (Oguntoyinbo and Goyal 2024).

### 4.1 Genomic Imprinting

Genomic imprinting is an epigenetic mechanism in which gene expression is regulated based on parent‐of‐origin (MacDonald and Mann 2020). Imprinted genes are typically found in defined chromatin domains containing at least three genes, often including an lncRNA (Thamban et al. 2020). These regions are directed by Imprint Control Regions (ICRs), which are CpG‐rich and differentially methylated during gametogenesis in a parent‐specific manner (Noordermeer and Feil 2020). After fertilization, these methylation marks are maintained in somatic cells, ensuring monoallelic expression (Llères et al. 2021).

Imprinted lncRNAs are key regulators of ICRs. When the ICR is unmethylated, the lncRNA is transcribed and retained at the locus where it silences neighboring genes in cis (MacDonald and Mann 2020). One mechanism is transcriptional interference, in which the activity of the lncRNA's promoter disrupts nearby promoters on the same chromosome (Shearwin et al. 2005). For example, the maternally imprinted gene Dlk1, which encodes a Notch1‐inhibiting ligand (Grassi and Pietras 2022) is repressed during neural differentiation by the lncRNA Meg3. Meg3 accumulates at the locus and overlaps the Dlk1 promoter, thereby inhibiting its transcription (Sanli et al. 2018).

Similar mechanisms have been described for other imprinted lncRNAs, including UBE3A‐AST (Hsiao et al. 2019), NESPAS (Tibbit et al. 2015), and AIRN (Andergassen et al. 2019). In the case of Meg3 and AIRN, silencing involves not only transcriptional interference but also recruitment of PRC2, which deposits repressive histone marks on neighboring genes (Andergassen et al. 2019), indicating that imprinted lncRNAs coordinate both transcriptional and chromatin‐level gene repression.

The large genomic distances encompassed by ICRs require additional factors to modulate chromatin architecture at such a scale. One key player is CTCF, imprinted lncRNAs may help maintain CTCF‐binding site accessibility by sustaining promoter activity, as CTCF preferentially binds unmethylated DNA (Llères et al. 2021). A well‐characterized example is the H19 locus: upon CTCF recruital, H19 facilitates chromatin looping that brings distant regions into proximity, insulating the Igf2r promoter from its enhancers and guiding its paternal repression (Llères et al. 2019). Multiple TFs have been associated with mammalian imprinting, such as ZNF791 (Ahn et al. 2025), ZPF57 (Monteagudo‐Sánchez et al. 2020), and DNMT1 (Das et al. 2013).

### 4.2 Sex Chromosome Dosage Compensation

In vertebrates, the two most common sex chromosome systems (XY and ZW) are heteromorphic, meaning that one of the sex chromosomes has undergone degeneration. As a result, the heterogametic sex carries only a single copy of genes that are present in duplicate in the homogametic sex, leading to gene dosage imbalance (Livernois et al. 2012). LncRNAs play a central role in compensating for this imbalance: in eutherian mammals, dosage compensation is mediated by XIST, whereas in metatherians it is achieved through RSX. Although XIST and RSX evolved independently (Navarro‐Cobos and Brown 2025), they modulate a set of overlapping protein–protein interaction networks (McIntyre et al. 2024), likely mediated by analogous structural features (Sprague et al. 2019). This common regulatory mechanism may have evolved in the ancestor of therian mammals from a common pool of non‐coding transcripts that could have served as raw material for the evolution of both regulatory mechanisms (Waters et al. 2018). And is theorized that this toolbox may have been the starting point for independently evolved compensation mechanisms in fish or reptiles (Graves 2015).

DNA methylation patterns are conserved across vertebrates in autosomal gene regulatory regions, while sex chromosomes exhibit lineage‐specific methylation signatures—such as the potential involvement of miRNAs in male chicken Z chromosomes (Al Adhami et al. 2022). Nonetheless, dosage compensation in vertebrates remains an open question, and further investigation into these mechanisms across lineages will deepen our understanding of lncRNAs as key regulators of gene expression.

### 4.3 Pluripotency

PSCs have the capacity of self‐renewal under a complex regulatory network of TFs, signaling pathways, and epigenetic regulators (Hunkler et al. 2022). The PSCs regulatory network relies heavily on lncRNAs to maintain pluripotency; some, like LincQ, Gas5, and Panct1, interact with core pluripotency TFs (e.g., OCT4, SOX2, and NANOG) (Jing et al. 2020; Tu et al. 2018). Others recruit epigenetic regulators to specific genomic loci; for example, LncPRESS1 enhances reprogramming by sequestering the histone deacetylase SIRT6, preserving high histone acetylation (Jain et al. 2016). Additional mechanisms include modulating chromatin architecture (Du et al. 2021), and regulating other ncRNAs (Ghosh and Som 2024).

### 4.4 Differentiation and Organogenesis

During development PSCs give place to all the cellular lineages that form an organism through a highly orchestrated process termed differentiation. Temporal analyses during neurogenesis have uncovered complex lncRNA expression profiles and their functional interplay with key TFs (Kuruş et al. 2021). For example, mammalian lncRNA RUS mediates neural development through recruitment of TFs Phb, NOCL2, and Nup98 to different loci, acting as a central regulator promoting and repressing gene expression (Schneider et al. 2022).

Epigenomic studies using chromatin capture techniques highlighted the dynamic reorganization of chromatin during cardiogenesis, marked by the activation of > 170,000 enhancer regions (Vanoudenhove et al. 2020). These changes are accompanied by tightly coordinated lncRNAs, TFs, and miRNAs networks that modulate stage‐specific cardiac development (Li, Yan, et al. 2023).

While most pluripotency and differentiation studies have focused on mouse Embryonic Stem Cells, growing evidence from other species offers important insights. In zebrafish ( *Danio rerio* ), thousands of lncRNAs are differentially expressed during neurogenesis under both normal and stress conditions (Zhou et al. 2024) and functional analyses have revealed critical roles for these lncRNAs: CRISPR/Cas9 knockout of SlincR disrupted developmental and metabolic pathways and abolished caudal fin regeneration (Dasgupta et al. 2023). Similarly, MALAT1, which is highly expressed across tissues, regulates transcription factors such as GATA4 and EGR1, and its knockdown impairs neural development (Wu et al. 2019).

In chickens, skeletal muscle development involves diverse myofibres essential for growth and movement, with around 50 lncRNAs actively transcribed during this process. Notably, lncRNA MYH1G promotes myogenesis by recruiting TFs such as CDK1A/B, MYF5/6, and MYOG (Cai et al. 2024).

In amphibians, most developmental transcriptomic studies have focused on PCGs expression patterns on plasticity (Degani and Meerson 2024; Stuckert et al. 2023), tissue‐specificity (Ma et al. 2018; Richardson et al. 2018), and regeneration (Mahapatra et al. 2023; Matsunami et al. 2019). However, increasing evidence shows that lncRNAs could play important regulatory roles, with numerous transcripts now annotated across amphibian species (Arenas Gómez et al. 2018; Bai et al. 2021).

### 4.5 Metamorphosis

#### 4.5.1 HPT‐Axis

In amphibians, metamorphosis is controlled by the Hypothalamic–Pituitary–Thyroid (HPT) neuroendocrine axis, which regulates the synthesis and release of thyroid hormones (THs). The HPT axis is composed of thyrotropin‐releasing hormone (TRH)–producing neurons in the hypothalamus, thyroid‐stimulating hormone (TSH)‐producing thyrotropes, and the thyroid gland (Feldt‐Rasmussen et al. 2021). TRH binds to its receptors in the pituitary, stimulating the synthesis and secretion of TSH. Circulating TSH then binds to its receptor in the thyroid gland, inducing the production and release of thyroxine (T4) and lower levels of triiodothyronine (T3). T4 serves primarily as the precursor of T3 and is converted into the active hormone by deiodinases in peripheral target tissues (Paul et al. 2022). Notably, this synthesis pathway extends beyond vertebrates; recent findings in the tunicate *Styela clava* confirm that thyroglobulin, the essential TH precursor, is conserved across the broader Chordata phylum (Zhang et al. 2026).

The role of the HPT axis during amphibian metamorphosis is relatively well understood—see, for example, the work of Fort et al. (2007) and Paul et al. (2022). Nonetheless, how lncRNAs regulate or interact with this endocrine signaling pathway remains largely unclear. Evidence from other vertebrate systems suggests that lncRNAs are associated with HPT‐axis activity during pregnancy in pigs (Makowczenko et al. 2023), with the regulation of seasonal reproduction in sheep (La et al. 2020), and that alterations in HPT‐associated lncRNAs can lead to neuronal signaling changes affecting neurotransmission, metabolism, and immune responses (Oh et al. 2025).

Taken together, these findings suggest that the relationship between lncRNAs and the HPT axis is biologically significant. Therefore, investigating this association during amphibian metamorphosis—and in other developmental or physiological models—may provide important insights into the fine‐tuning of gene regulation underlying endocrine‐controlled transitions such as metamorphosis.

#### 4.5.2 Thyroid Hormone Signaling

T3 orchestrates the maturation of multiple organs into their adult form during post‐embryonic development, a period encompassing the final stages of gestation and extending into early postnatal life in mammals (Fu et al. 2017), as well as metamorphosis in anurans (Shibata et al. 2021). T3 exerts its effects by binding to nuclear thyroid hormone receptors (TRs), of which two conserved isoforms, TRα and TRβ, are present across vertebrates (Paul et al. 2022). TRs form heterodimers with 9‐cis retinoic acid receptors (RXRs) and bind to thyroid hormone response elements (TREs) in DNA, where they function as transcriptional repressors in the absence of ligand (Wen et al. 2017). Upon T3 binding, TRs undergo a conformational change that displaces co‐repressors and recruits co‐activators, thereby eliciting the transcription of target genes that drive the morphological, biochemical, and physiological changes associated with metamorphosis (Zhao, Liu, et al. 2016).

Genome‐wide TRE probing in *Xenopus tropicalis* , showed that T3 induces active transcription of a small subset of genes that act as global regulators of downstream signaling pathways during metamorphosis (Fu et al. 2017). Around 40% of TREs are present outside transcription start sites of PCGs, particularly in intergenic regions (Raj et al. 2023), suggesting that transcription of non‐coding RNAs is initiated by T3 during amphibian metamorphosis.

High‐throughput sequencing (HTS) has uncovered thousands of unique lncRNAs potentially involved in metamorphic regulation. In *Lithobates catesbeiana*, about 6000 lncRNA genes were identified, of which ~1000 were differentially expressed during metamorphosis (Birol et al. 2015). Even larger numbers—over 50,000 putative lncRNAs—have been reported in whole‐larvae transcriptomes of *Rhinella arenarum* (Ceschin et al. 2020), and *Pelobates cultripes* (Liedtke et al. 2022). Tissue‐specific studies, such as those on dorsal muscle remodeling in *Microhyla fissipes* , identified more than 16,000 differentially expressed lncRNAs during metamorphic transitions (Liu et al. 2023).

Although the biological functions of lncRNAs during metamorphosis remain largely unexplored, emerging data offer initial insights. In *Xenopus laevis* , lncRNAs are actively expressed during testis development and respond to endocrine disruptors, correlating with PCGs involved in Wnt signaling, autophagy, cell cycle, and metabolism (Qi et al. 2021; Zhang, Sai, et al. 2023). In *Salamandra salamandra* and *Ommatotriton vittatus* , metamorphosis‐associated transcriptomes have been analyzed, but only for PCGs, revealing transcriptional changes tied to morphophysiological transformations (Degani and Meerson 2024; Sanchez et al. 2018). The absence of lncRNA annotation in these studies highlights a significant opportunity to re‐explore public datasets to investigate lncRNA function in Caudata.

In other vertebrates where metamorphosis is less accessible, post‐embryonic transcriptomics have identified thousands of lncRNAs correlated with growth and development. For example, in pre‐weaning calves, ~4000 lncRNAs were linked to post‐transcriptional regulation (Ibeagha‐Awemu et al. 2018), while in chickens, stage‐specific lncRNA–mRNA networks were implicated in embryonic development, cell proliferation, and tissue formation (Li et al. 2017). Whether these patterns are regulated by THs, as in amphibians, remains an open question.

#### 4.5.3 Limb Development

Vertebrate limbs form through a conserved induction, patterning, and differentiation mechanisms, with lineage‐specific modifications to the underlying molecular programs (Young and Tabin 2017). Limb development initiates in the lateral plate mesoderm, forming a bud composed of a mesenchymal core covered by an ectodermal epithelium, where growth factors such as FGF10 and FGF8 trigger limb initiation and sustain outgrowth (Royle et al. 2021). Subsequently, the limb bud undergoes a tightly regulated patterning process in which positional information is established through the coordinated activity of HOX genes, the canonical WNT signaling pathway, and additional TFs (Zuniga and Zeller 2020). Finally, spatially organized gene expression gradients guide morphogenesis and tissue differentiation, giving rise to the final structure of the limb (Thompson 2021).

Upstream of limb bud induction, limb positioning is regulated by transcription factors such as Tbx5, which plays a central role in forelimb specification (Moreau et al. 2019). LncRNAs have been associated to this regulatory landscape; for example, transcriptomic analyses of bat wing development identified approximately 190 lncRNAs that are differentially expressed during limb development, with two transcripts—HOTTIP and Tbx5‐as1—showing strong correlation with Tbx5 expression and proper limb formation (Eckalbar et al. 2016). Additional lncRNAs have been reported to interact with Tbx5‐associated pathways in other biological contexts, including bone formation (Kcnq1ot1) (Wang et al. 2022), cardiac hypertrophy (Sngh7) (Zhang, Song, et al. 2023), or cardiac atrial remodeling (FOG2) (Broman et al. 2024). Together, these observations suggest that lncRNAs may participate in regulatory networks involving Tbx5, highlighting the need for experimental validation and the identification of additional molecular players during limb development.

Hox genes are highly conserved TFs that play essential roles in establishing cell identity and morphology along major developmental axes, such as the anteroposterior and proximodistal axes, through tightly regulated spatiotemporal expression patterns that reflect their genomic organization (Hubert and Wellik 2023). Regulation of HOX gene expression occurs through the formation of TADs, which enable coordinated waves of transcription required for proper limb patterning and development. This process is, in part, orchestrated by the lncRNA HOTTIP, which mediates chromatin interactions within the HOXA cluster (Luo et al. 2020; Rodríguez‐Carballo et al. 2020).

Notably, the regulatory network underlying limb development appears to be evolutionarily conserved. Orthologues of the lncRNAs HOXA‐AS3 and HOXB‐AS3 have been identified and functionally validated across multiple vertebrate lineages, where they display conserved expression patterns and regulatory roles (Degani et al. 2021). Another lncRNA, HOTAIRM1, is present in vertebrates as early as amphioxus and conserved in *Xenopus*; it is located adjacent to the HOX cluster, and its depletion results in a lethal phenotype (Herrera‐Úbeda et al. 2019). Together, these findings suggest that lncRNA‐mediated regulation of HOX clusters during limb development is both functionally essential and evolutionarily conserved (Leite‐Castro et al. 2016). Further identification of additional HOX‐associated lncRNAs may reveal a more complex regulatory network, while functional validation of these transcripts will be critical to determine the extent and mechanisms of their roles and conservation across vertebrates.

#### 4.5.4 Intestine Remodeling

During amphibian metamorphosis, the intestine undergoes TH–dependent remodeling to adapt from an aquatic herbivorous to a terrestrial carnivorous lifestyle. This process involves shortening of the small intestine, development of intestinal folds, and establishment of a self‐renewing adult epithelium that supports efficient digestion and nutrient absorption (Ishizuya‐Oka and Hasebe 2013). Intestinal remodeling occurs between limb development and tail resorption and is characterized by the coordinated degeneration of the larval epithelium through apoptosis and de novo differentiation and proliferation of adult epithelial stem cells (Paul et al. 2022).

In mammalian systems, lncRNAs have been implicated in both normal and pathological regulation of the intestinal barrier, where they modulate processes such as apoptosis, cell proliferation, and inflammatory responses (Chen et al. 2018; Chen, Zhang, et al. 2021). However, to the best of our knowledge, no lncRNA‐focused analyses have been performed during intestinal remodeling in amphibian models. Nevertheless, potential regulatory roles for lncRNAs may be inferred by examining the gene expression programs underlying this process, which include metalloproteinases (MMP2, MMP9, MMP11, MMP13, and MMP14), apoptotic regulators (caspase‐3, caspase‐9, and BAX), adult intestinal stem cell markers (XHH and OLFM4), and differentiation‐associated genes such as ror2 and wnt5 (Shibata et al. 2021).

LncRNAs have been found associated with metalloproteinases in clinical context such as a direct correlation between lncRNA GAS5 (Lucafò et al. 2019) and lncRNA PVT1 (Zhang et al. 2019) with MMP2 and MMP9. Additionally, lncRNAs have been shown to regulate intestinal epithelial apoptosis (Yan et al. 2024), and stem cell differentiation downstream of Wnt signaling pathways (Schwarzmueller et al. 2025). While assuming conservation of these mechanisms across vertebrate lineages would be premature, these findings provide a valuable starting framework for investigating the contribution of lncRNAs to intestinal remodeling in amphibians and for exploring their potential evolutionary conservation.

#### 4.5.5 Tail Resorption

Tail resorption is one of the most dramatic morphological changes during amphibian metamorphosis and occurs shortly after the peak accumulation of T3 at Nieuwkoop–Faber stage 62 (Nakajima et al. 2019). This process follows the so‐called murder/suicide model, in which TH induces fibroblasts to secrete metalloproteinases that degrade the extracellular matrix (ECM), leading to the death of neighboring cells (“murder”). Concurrently, TH accumulation directly activates apoptotic pathways in myoblasts, resulting in autonomous cell death—“suicide” (Nakai et al. 2017).

This model has been extensively studied, and its endocrine regulation is now relatively well characterized (Buchholz 2017; Buchholz and Shi 2018; Nakajima et al. 2018; Sakane et al. 2018). Nonetheless, our understanding of the gene expression programs underlying tail resorption remains limited. Only recently have transcriptomic approaches begun to reveal the molecular players and regulatory pathways involved in this dramatic developmental transformation (Liu et al. 2023; Nakajima et al. 2020; Wang et al. 2019; Zhao, Liu, et al. 2016).

Although the role lncRNAs in amphibian metamorphosis remains unexplored, lncRNAs have been implicated in several biological processes that are central to tail reabsorption, including apoptosis—particularly in cancer cell models—(Guo et al. 2024; Hussain et al. 2023; Pang et al. 2023), ECM degradation (Chen, Zhu, et al. 2021; Fan et al. 2022; Wu et al. 2022), and autophagy (Jiang et al. 2023; Lei et al. 2023; Liang et al. 2024). Together, these observations indicate that lncRNAs act as important regulators of these processes in other biological contexts, allowing us to theorize that they may constitute an additional, yet unexplored, layer of regulation during tail resorption.

#### 4.5.6 Metamorphosis‐Associated Signaling Pathways

T3 signaling during metamorphosis activates several downstream pathways that coordinate tissue transformation, including Wnt/β‐catenin, Notch, Hedgehog, and BMP/TGF‐β signaling. In amphibians, these pathways regulate organogenesis, cell‐cycle progression, and proliferation in multiple tissues, such as the hindlimb (Tanizaki et al. 2022), liver (Tanizaki, Wang, et al. 2023), intestinal epithelium (Hasebe et al. 2016), and adult stem cell formation and differentiation (Ishizuya‐Oka et al. 2014).

In the absence of Wnt stimulation, cytoplasmic β‐catenin is continuously targeted for proteasomal degradation by the destruction complex composed of APC, Axin, and GSK3β. Upon Wnt ligand binding to its membrane receptors (Frizzled and LRP5/6), the destruction complex is functionally inactivated through recruitment of Axin to the membrane, preventing β‐catenin phosphorylation and degradation. Stabilized β‐catenin accumulates in the cytoplasm and subsequently translocate to the nucleus, where it associates with TCF/LEF, converting them from transcriptional repressors into activators of Wnt target genes (Liu, Xiao, et al. 2022). There is a comprehensive cross‐talk between lncRNAs and Wnt signaling pathway revised in Zarkou et al. (2018).

Notch signaling mediates short‐range cell–cell communication and functions as a binary cell‐fate determinant, enabling cells to select between pre‐existing developmental programs in a context‐dependent manner. Activation occurs when a membrane‐bound Notch ligand (Delta or Jagged) on one cell binds to the Notch receptor on an adjacent cell. This interaction triggers sequential proteolytic cleavages of the receptor, culminating in γ‐secretase–mediated release of the Notch intracellular domain (NICD). The NICD translocate to the nucleus, where it associates with the DNA‐binding protein CSL (CBF1/RBPJκ in vertebrates), converting it from a transcriptional repressor into an activator complex that induces expression of downstream target genes (Zhou et al. 2022).

Given the central role of Notch signaling in regulating cell fate decisions, and the emerging importance of lncRNAs as modulators of gene expression, accumulating evidence highlights a functional interplay with lncRNAs. This relationship has been extensively documented in cancer (Siddique et al. 2024), tissue homeostasis (Salviano‐Silva et al. 2020), cell division (Chen and Wu 2025), and adaptive immunity (Zeni and Mraz 2021). However, its potential contribution to amphibian metamorphosis remains largely unexplored, representing an important opportunity to understand how lncRNAs may fine‐tune developmental signaling during large‐scale tissue remodeling.

Among signaling pathways, the Transforming Growth Factor β (TGF‐β) pathway is highly conserved across the animal kingdom. Throughout metazoans, it regulates a wide range of embryonic and adult processes, enabling tissue‐specific control of cell metabolism, growth, proliferation, survival, adhesion, migration, and apoptosis. Its activity is essential for maintaining tissue homeostasis under physiological conditions (Tzavlaki and Moustakas 2020).

TGF‐β is synthesized in the endoplasmic reticulum as a latent precursor that forms a homodimer. The dimer then transits through the Golgi apparatus, where it is cleaved by a furin‐like protease into the mature cytokine and the latency‐associated peptide (LAP). These components remain non‐covalently associated, forming the small latent complex, which is secreted into the extracellular matrix. There, it can associate with latent TGF‐β binding proteins (LTBPs), forming a large latent complex that is deposited in the matrix. Activation of TGF‐β requires release of the mature cytokine from this complex, a process mediated by factors such as proteases, acidic or basic pH, thrombospondin‐1, and integrins (Deng et al. 2024).

TGF‐B has been identified as a key signaling pathway in metamorphosis of different vertebrates including *Xenopus* (Das et al. 2006), flatfish (Louro et al. 2020), and sea lamprey (Chung‐Davidson et al. 2020). Additionally, homologues of the TGF‐β superfamily have been identified in the Paridae family (Order Passeriformes) (Sultana et al. 2025).

Evidence linking lncRNAs to TGF‐β signaling during embryonic development further supports its regulatory complexity. For example, lncRNA‐Smad7 regulates cell fate determination in mouse embryonic stem cells (mESCs) by downregulating *Bmp2*, thereby impairing cardiomyocyte differentiation (Kong et al. 2022). Similarly, during sex differentiation, a network of 11 lncRNAs orchestrates germ cell differentiation through regulation of TGF‐β and other signaling pathways (Gao et al. 2020). Whether lncRNA‐mediated regulation of TGF‐β extends from embryogenesis into later developmental stages such as metamorphosis is still to be determined, making it a promising avenue for further research.

#### 4.5.7 Metamorphosis‐Associated lncRNAs in Other Organisms

Metamorphosis is an ancestral life‐history trait present across multiple invertebrate phyla and at the roots of vertebrates. In contrast, in insects, metamorphosis is considered a derived trait that emerged after terrestrial colonization and is regulated by endocrine mechanisms distinct from those operating in vertebrates (Truman 2019). Nonetheless, the relationship between lncRNAs and metamorphosis has been studied in insects, particularly in dipterans and lepidopterans.

*Drosophila melanogaster* is a powerful platform for functional genomic analyses because it's a model organism with low genetic redundancy, a high number of functional homologues to human genes—including lncRNAs—and evolutionarily conserved molecular pathways, (Li et al. 2019). During fly metamorphosis, the expression of a substantial proportion of lncRNAs (~40%) is under tight temporal regulation throughout the transformation process, suggesting a high degree of developmental specificity (Chen et al. 2016). Similar findings have been reported for lncRNAs involved in imaginal disc development during metamorphosis. These lncRNAs display significantly higher tissue‐ and stage‐specific expression patterns compared with PCGs, further supporting the idea that lncRNAs may play regulatory roles during metamorphosis (Camilleri‐Robles et al. 2024).

In the flesh fly (*Sarcophaga peregrina*), lncRNAs are likewise differentially expressed in a stage‐specific manner during development. These lncRNAs are associated with enriched biological processes relevant to insect metamorphosis, including ventral midline development, hindgut morphogenesis, and compound eye development. Bioinformatic analyses suggest that they may exert regulatory effects through pathways such as Hedgehog signaling, as well as metabolic and biosynthetic processes (Shang et al. 2023). Similar results were reported for *Aedes albopictus* , where around 500 lncRNAs are correlated with metamorphosis potentially regulating clusters of PCGs responsible for chitin metabolism, peptidase activity and hormone production (Liu, Cheng, et al. 2022).

LncRNAs not only participate in insect metamorphosis but may actively regulate it by modulating metamorphosis‐associated PCGs. This hypothesis is supported by observations in diamondback moth (*Plutella xyllostela*) where lncRNA expression patterns correlate with metamorphosis‐associated PCGs whose loci either partially overlap with or are in close genomic proximity to the corresponding lncRNA (Liu et al. 2017).

Functional evidence further supports their regulatory role. For example, in silkworm ( *Bombyx mori* ) the let‐7 miRNA cluster is transcribed from the last exon of lncR17454 whose knockdown arrests metamorphosis by altering the expression of genes downstream of the ecdysone signaling pathway. Importantly, rescue experiments restore normal development, confirming the functional relevance of this lncRNA during metamorphosis (Fu et al. 2022).

Despite growing evidence of their involvement, the functional roles of lncRNAs during insect metamorphosis remain largely unresolved. Transcriptomic studies report extensive differential expression of lncRNAs during metamorphic transitions, with predicted target genes enriched in pathways related to autophagy, chitin biosynthesis, and metabolic remodeling (Jia et al. 2023; Qiao et al. 2021). Making it a great opportunity for further research and experimental validation.

The relationship between lncRNAs and metamorphosis extends across diverse marine invertebrate phyla. In the sponge *Amphimedon queenslandica*, lncRNAs are involved during larval settlement prior to metamorphosis (Say and Degnan 2020). Similarly to their bilaterian counterparts, *A. queenslandica* 's lncRNAs exhibit tissue‐ and stage‐specific expression patterns during embryogenesis, larval development, and metamorphosis. These expression profiles suggest potential regulatory roles in key developmental processes, including cell proliferation, tissue morphogenesis, and Wnt signaling pathways (Gaiti et al. 2015). However, their precise functional mechanisms remain to be elucidated.

In the coral *Acropora digitifera* (Cnidaria), temporally regulated lncRNA expression patterns associated with metamorphosis have been identified and proposed to function as regulators of this process (Reyes‐Bermudez et al. 2016). Similarly, in Mollusca, up to 47 lncRNAs have been suggested to participate in larval settlement and metamorphosis of the Pacific oyster *Crassostrea gigas* (Yu et al. 2016). Similar findings were reported in the tunicate *Ciona savignyi* (Urochordata), where experimental validation revealed lncRNAs with distinct differential expression patterns between early and late metamorphic stages, suggesting regulatory roles in processes such as initial notochord lumen formation and subsequent lumen expansion (Wei and Dong 2018). In echinoderms, approximately 5000 high‐confidence lncRNAs were annotated during larval development of *Paracentrotus lividus* ; however, their direct involvement in metamorphosis was not specifically evaluated (Marlétaz et al. 2023).

Overall, these studies indicate that across the tree of life there is a strong association between lncRNAs and metamorphosis. Despite this emerging pattern, the mechanistic roles of lncRNAs during this critical life‐cycle transition remain poorly understood. Further research in diverse model and non‐model organisms will be essential to uncover conserved regulatory pathways and shared molecular processes underlying metamorphosis.

## 5 Bioinformatic Strategies to Identify and Characterize lncRNAs

Advances in HTS technologies have made it increasingly feasible to study entire transcriptomes, enabling researchers to characterize an organism's full gene repertoire and analyze gene expression changes across biological contexts to generate hypotheses about gene regulation (Raghavan et al. 2022). To accomplish this, most studies rely on RNA sequencing (RNA‐seq); however, to extract biologically meaningful information, raw sequence reads must first be assembled and annotated into transcripts (Harris et al. 2017). Annotation can be achieved either through genome‐guided approaches, in which reads are mapped to a reference genome to reconstruct their transcripts of origin, or through de novo (reference‐free) assembly when no genome is available and transcript reconstruction relies solely on information contained within the reads themselves (Raghavan et al. 2022).

When focusing on lncRNAs, the complexity of transcriptome analysis increases substantially. Despite the growing number of annotated lncRNA transcripts, only a small fraction has been experimentally validated or manually curated, and most validated examples originate from well‐established model organisms (Adjeroh et al. 2024). Furthermore, many early computational tools were originally designed to classify PCGs and therefore may not adequately capture the structural and functional diversity characteristic of lncRNAs, which differ markedly from mRNAs (Cao et al. 2018). To address these limitations, a wide range of specialized computational tools and databases has been developed to improve lncRNA identification and to predict their potential biological functions. In this section, we compile and examine current databases and in silico resources that facilitate the functional characterization of lncRNAs both de novo and when high quality references are available.

### 5.1 De Novo LncRNA Identification

In silico tools have been developed to assess the coding potential of raw sequences, enabling the distinction between lncRNAs and mRNAs based on differences in coding capacity and nucleotide usage bias (Pinkney et al. 2020). These tools employ machine learning algorithms to predict coding probabilities and can be broadly categorized into four main types based on their underlying methodology: Support Vector Machines (SVMs), Random Forests (RFs), Logistic Regression (LRs), and Deep Learning (DL) techniques. A summary of the tools can be found in Table 1.

**TABLE 1:** Summary of lncRNA identification tools.

Tool	Algorithm	Features	References	Link
CPC2	SVM	ORF length and integrity, Ficket TESTCODE, hexamer usage bias	(Kang et al. 2017)	https://github.com/gao‐lab/CPC2_standalone
PLEK2	SVM	K‐mer frequencies, calibrated ORF features	(Li et al. 2024)	https://sourceforge.net/projects/plek2/
LongDist	SVM	Distance based features from k‐mers (composition based)	(Schneider et al. 2017)	https://github.com/hugowschneider/longdist.py
FEELnc	RF	Multi k‐mer frequencies, ORF‐related features	(Wucher et al. 2017)	https://github.com/tderrien/FEELnc
LncRNA‐ID	RF	ORF‐features, coding composition features, protein‐related features, evolutionary conservation	(Achawanantakun et al. 2015)	https://github.com/zhangy72/LncRNA‐ID
CPAT3	LR	ORF size and coverage, Ficket TESTCODE, hexamer usage bias	(Wang et al. 2013)	https://github.com/liguowang/cpat
LncScore	LR	ORF‐features, hexamer usage bias, Ficket TESTCODE, GC content, transcript length	(Zhao, Song, and Wang 2016)	https://github.com/WGLab/lncScore
LncADeep	DL	ORF‐features, coding potential features, protein‐related features, homology‐based features	(Yang et al. 2018)	https://github.com/cyang235/LncADeep
DeepLnc	DL	K‐mer frequencies, ORF‐related features, GC content	(Tripathi et al. 2016)	https://bioserver.iiita.ac.in/deeplnc (currently offline 27/02/26)

*Note:* This table presents the name of each tool, the classification algorithm employed, the features used to distinguish lncRNAs from protein‐coding transcripts, and the corresponding repository link where the software can be accessed.

#### 5.1.1 Support Vector Machines

SVMs are used to classify variables into binary outcomes by constructing a decision boundary, or hyperplane, which separates the data into classes (Pisner and Schnyer 2019). SVMs maximize the hyperplane relying on specific data points known as support vectors (Joshi 2023), enhancing generalization across different datasets and scenarios (Wang 2022). SVMs are robust classifiers that balance specificity and sensibility, and are easy to access and use, but they tend to have limited tolerance to heterogenous assemblies or short reads. The most popular tool: Coding Potential Calculator 2 (CPC2) (Kang et al. 2017) assigns a coding probability to each transcript using a combination of nucleotide composition bias (Fickett TESTCODE), open reading frame (ORF) length and integrity, and the stability of putative peptides (Zheng et al. 2021). LongDist (Schneider et al. 2017) uses nucleotide patterns and ORF characteristics to distinguish lncRNAs from PCGs. Alternatively, PLEK2 (Li et al. 2024) uses *k‐mer* pattern with a sliding window and calibrated ORF length to train its SVM.

#### 5.1.2 Random Forest

RFs generate multiple decision trees using bootstrapped subsets of the data and aggregate their predictions—typically by averaging or majority voting—to produce a result (Ahmed Salman et al. 2024), making them robust models with high accuracy and strong performance even with small sample sizes or high‐dimensional data (Biau and Scornet 2015). For instance, FEELnc (FlExible Extraction of lncRNAs) (Wucher et al. 2017) integrates a relaxed ORF detector—based on integrity, size, and coverage—with a dynamic k‐mer frequency counter (ranging from 1 to 12) to calculate a coding probability score between 0 (non‐coding) and 1 (coding). Alternatively, LncRNA‐ID (Achawanantakun et al. 2015) incorporates features such as ORF coverage and length, Kozak‐motif presence, ribosome foot printing data, and homology with known protein domains to train a balanced RF model. This approach compensates for the disproportionate ratio of coding and non‐coding transcripts, enhancing its predictive performance. Despite their advantages, RF models are prone to overfitting in datasets with high levels of noise or when dominant predictive features carry excessively polarized weight values.

#### 5.1.3 Logistic Regression

LR describes the relationship between a binary response variable and one or more independent variables (covariates) (Harris 2021). The model assumes a continuous distribution of the outcome by modeling the natural logarithm of the odds (Zabor et al. 2022). One of LR's main strengths lies in the interpretability of its coefficients: by exponentiating the slope, it becomes an odds ratio, representing the change in odds for a one‐unit increase in the predictor variable (Schober and Vetter 2021). This interpretability and predictive accuracy, make LRs a valuable tool in many biological applications, including coding potential estimation. Coding Potential Assessment Tool (CPAT) (Wang et al. 2013), uses an LR to calculate the coding probability of transcripts based on ORF size and coverage, Fickett TESTCODE, and hexamer usage bias. CPAT includes pre‐trained algorithms for model species and provides guidance for retraining the model using custom datasets. Similarly, LncScore (Zhao, Song, and Wang 2016), employs LR by incorporating six ORF‐related features, hexamer usage bias, and exonic features, making it especially effective for species with high‐quality genome annotations.

#### 5.1.4 Deep Learning

DL a subset of machine learning, leverages artificial neural networks to process large, unstructured datasets by mimicking brain‐like information flow (Sharifani and Amini 2023). These models use multiple hierarchical layers—input, hidden, and output—to extract increasingly complex features, making them especially powerful for lncRNA prediction (Mathew et al. 2021). LncADeep utilizes features like ORF length, hexamer score, GC content, Fickett score, and sequence entropy within a Deep Belief Network to distinguish lncRNAs from mRNAs, with the added benefit of handling partial transcripts (Yang et al. 2018). Similarly, DeepLnc identifies characteristic k‐mer usage patterns through a deep neural network, using Shannon entropy to optimize memory efficiency and reduce redundancy (Tripathi et al. 2016).

DL tools offer high predictive accuracy without requiring extensive manual feature selection. However, they rely on large training datasets (Mathew et al. 2021), often act as “black boxes” due to limited interpretability of internal decision making (Sharifani and Amini 2023), and require substantial computational resources, limiting their accessibility to users without high‐performance computing.

Benchmarking studies reveal that many lncRNA prediction tools perform with comparable accuracy, though results depend on factors such as the origin of transcript data, sequence quality, and the availability of ancillary information (Ramakrishnaiah et al. 2020; Zheng et al. 2021). Since no single tool consistently outperforms others, combining multiple methodologies is recommended to increase stringency and reliability in lncRNA identification (Duan et al. 2021).

### 5.2 Annotated lncRNAs

Multiple databases annotate lncRNAs with their known functions in specific biological scenarios such as cancer (Gao et al. 2021), diseases and immune response (Lin et al. 2024), tissue‐specific expression (Li, Liu, et al. 2021), sub‐cellular localization (Mas‐Ponte et al. 2017), miRNA interactions (Zhao et al. 2023) and protein interactions (Zheng, Luo, et al. 2023). When analyzing large sets of lncRNAs in complex processes integrating information from multiple sources can become burdensome. To address this, “metabases” that integrate data from various repositories have emerged (Pinkney et al. 2020).

#### 5.2.1 LNCipedia

LNCipedia compiles lncRNAs from Ensembl (Harrison et al. 2024) a genome annotation platform for 1000 species across the tree of life, RefSeq (Goldfarb et al. 2025), NCBI's curated reference set for genomic and transcriptomic sequences from about 2000 eukaryotic species, FANTOM‐CAT (Hon et al. 2017), transcriptomic atlas of 27,000 human lncRNAs across 1800 tissues, and NONCODE (Zhao et al. 2021), a dedicated repository for non‐coding RNAs from 16 animal species. LNCipedia latest release (v5.2) includes over 127,000 unique transcripts and more than 56,000 genes (Volders et al. 2019). Beyond sequence curation, LNCipedia assigns entries with literature‐based functional annotations and assigns stable identifiers, making it especially useful for cross‐referencing known functional roles.

#### 5.2.2 LncBook

LncBook is a systematic lncRNA annotation generated from multi‐omics data (Li, Liu, et al. 2023): a human reference transcriptome with over 77,000 transcripts across 30 normal tissues and 18 tumors (RefLnc) (Jiang et al. 2019), GENCODE (genomic annotation for mouse and human) (Frankish et al. 2023), FANTOM‐CAT, NONCODE, and independent experimental validations. In its latest version (v2.0), it features over 95,000 lncRNAs, with disease‐variant associations, methylation profiles, expression patterns from multiple human tissues, and predicted interactions with proteins and miRNAs. Overall, LncBook is one of the most comprehensive repositories for human lncRNAs, offering a user‐friendly web interface for browsing, visualization, and functional analysis.

### 5.3 Functional Annotation

Having identified lncRNAs, the next step is assigning potential functional roles. LncRNA annotation can be approached through two strategies: searching known roles in lncRNA databases, or de novo identification of functional roles.

#### 5.3.1 Expression Analyses

Analyzing lncRNA expression by quantifying read alignments through genome‐guided or reference‐free methods can provide key insights into lncRNA function. In genome‐guided approaches, transcripts are aligned to a reference genome to produce expression matrices. Tools like FeatureCounts assign reads based on genomic features such as exons, promoters, or intergenic regions (Liao et al. 2014) while HTS‐count focuses specifically on exonic reads (Anders et al. 2014). When a reference genome is unavailable, transcript‐level expression can be estimated using probabilistic alignment to a reference transcriptome (Yi et al. 2018). Salmon uses a quasi‐mapping algorithm that assigns reads via k‐mer equivalence classes and corrects for sequence‐specific biases (Patro et al. 2017). Kallisto performs pseudo‐alignment by indexing k‐mers and applying a likelihood‐based model to estimate transcript abundance (Bray et al. 2016).

These expression estimates support the identification of differential expression patterns underlying complex biological processes like metamorphosis. Tools such as EdgeR (Chen et al. 2025), and DESeq2 (Love et al. 2014) use negative binomial models to normalize counts, estimate dispersion, and perform hypothesis testing. Notably, the latest version of EdgeR supports fractional counts from tools like Salmon, making it ideal for modern workflows, while DESeq2's internal normalization is particularly effective for small datasets.

#### 5.3.2 Structure Prediction

The single‐stranded nature of lncRNAs allows them to self‐fold into diverse secondary structures that mediate interactions with nucleic acids and proteins. To predict these conformations, computational tools use thermodynamic and context‐dependent models.

Thermodynamic approaches estimate folding by minimizing free energy. Tools like RNAstructure (Reuter and Mathews 2010) analyze folding using enthalpy and free energy parameters, while RNAFold (Lorenz et al. 2011), calculates a partition function to identify the most probable structure among all possibilities. However, these tools are less accurate for long sequences and struggle with large datasets. To address this, recent algorithms like LinearFold offer up to 2000× faster performance, improving base‐pairing sensitivity for long RNAs (Zhang et al. 2020). Advanced models such as MXfold2 integrate deep learning to predict structures by combining neural network‐derived folding scores with thermodynamic parameters, optimizing predictions based on Maximum Expected Accuracy algorithms (Sato et al. 2021).

#### 5.3.3 Interaction Prediction

As lncRNAs regulate multiple molecular players, constructing regulatory networks by leveraging extensively annotated repositories of RNA‐binding motifs and lncRNA–protein interactions (LPI) have been the focus of current research (Philip et al. 2021). Publicly available datasets of RBPs such as POSTAR3 (Zhao et al. 2022), NPInter (Zheng, Luo, et al. 2023), and RNAInter (Kang et al. 2022) incorporate experimentally validated LPIs from multiple HTS assays across species, making them robust resources when working with annotated lncRNAs. These repositories are harnessed by LPI prediction tools to train powerful machine learning models and uncover novel potential interactions (Zhong et al. 2021). For instance, LPI‐SKF infers unknown interactions using similarity kernel fusion graphs (Zhou et al. 2020), while RPI‐SE (Yi et al. 2020) combines multiple machine learning algorithms to predict interactions based on the sequence features of both the lncRNA and the protein.

Several tools have been developed to predict lncRNA‐DNA interactions with recent advances enabling genome‐wide analyses. TriplexAligner (Warwick et al. 2022) employs a probabilistic nucleotide‐pairing model informed by experimental data and Hoogsteen base‐pairing rules to enhance triplex prediction accuracy. Fasim‐LongTarget (Wen et al. 2022) identifies multiple triplex‐forming sites by extensively applying Hoogsteen pairing rules to DNA sequences and supports parallelization, enabling accurate and efficient genome‐wide predictions.

## 6 Evolutionary Conservation of lncRNAs: Sequence, Structure, and Position

Approximately 70% of human lncRNAs are not detectable in species that diverged over 50 million years ago, which initially supported the notion that lncRNAs lack evolutionary conservation (Hezroni et al. 2015). However, emerging views propose that lncRNAs may serve as a reservoir for evolutionary innovation and rapid adaptation. Their rapid turnover—frequent emergence and loss—often stems from non‐functional genomic regions, where they can accumulate mutations without strong selective constraints and occasionally gain novel, beneficial functions (Kapusta and Feschotte 2014; Palazzo and Koonin 2020).

More recent studies have shown that certain lncRNAs in vertebrates are under stronger purifying selection than expected for random intergenic regions, and that their promoter and splicing sites often exhibit conservation levels comparable to those of PCGs (Chernikova et al. 2016). These findings suggest that lncRNAs may follow different evolutionary trajectories from those of PCGs, with some evolving rapidly while others are functionally constrained. Indeed, conservation in lncRNAs can be detected across three dimensions: primary sequence, secondary structure, and genomic positional conservation (synteny) (Nitsche and Stadler 2017).

### 6.1 Sequence Conservation

Although lncRNAs show low primary sequence conservation, many contain conserved microhomologies (~200 bp) (Quinn et al. 2016) and maintain tissue‐specific expression patterns, suggesting functional conservation despite sequence divergence (Ulitsky et al. 2011). Notably, NEAT1 is present in all tetrapods (except birds and squamates), while MALAT1 is conserved across gnathostomes, illustrating the deep evolutionary roots of some functionally important lncRNAs (Weghorst et al. 2024).

A subset of lncRNAs originates from Ultra‐Conserved Elements (UCEs) > 100 bp sequences with > 97% identity across mammals and 75%–90% across vertebrates (Cummins et al. 2024). About 60% of UCEs are transcribed into lncRNAs, many involved in neural development (Zhou et al. 2017). For example, Evf2 is transcribed during forebrain development, where it modulates enhancer‐TF interactions essential for interneuron diversity (Cajigas et al. 2018).

Some conserved lncRNAs retain their function across species: THOR, originally identified in humans, is conserved in zebrafish, where it regulates IGF2BP mRNA stability and affects fertilization (Hosono et al. 2017); similarly, TUNA contains a conserved motif regulating neural lineage commitment in all vertebrates, and human orthologues restore function in both cases (Lin et al. 2014).

However, sequence conservation does not always equal functional conservation. Cyrano is essential for zebrafish notochord development via a conserved domain (Ulitsky et al. 2011), but knockdown in human and mouse stem cells yields minimal phenotypic or transcriptional changes, suggesting divergent roles in mammals (Hunkler et al. 2020).

### 6.2 Positional Conservation

Synteny refers to homologous genes arranged in the same order on the same chromosome across species (Ranjan et al. 2025). In primates, ~5800 lncRNAs show syntenic conservation (Bryzghalov et al. 2020), while ~4200 human lncRNAs have putative syntenic orthologues in mouse, reflecting slightly reduced conservation over greater evolutionary distances (Foulkes et al. 2021).

Syntenic lncRNAs (syntologs) suggest that transcription from specific loci rather than the transcript itself may be functionally important (Szcześniak et al. 2021). Many regulate neighboring PCGs; ~500 syntologs conserved from zebrafish to humans are developmentally expressed (Ranjan et al. 2025). Cross‐species knockdown rescue experiments between human and zebrafish point to conserved function via overlapping RBPs interactomes (Huang et al. 2024). Some syntologs, like RUS, act in *trans* by recruiting chromatin regulators (Noc2L, Brd2, Smarca5) during neural development (Schneider et al. 2022).

Synteny analysis for lncRNAs has limitations: conserved regions may fall within synteny blocks of PCGs, and reliable detection depends on high‐quality genomic annotations which are lacking for many species of non‐model vertebrates (Szcześniak et al. 2021).

### 6.3 Structural Conservation

The three‐dimensional structure of lncRNAs tends to be more dynamic compared with mRNAs, whose structure is constrained by the amino acid sequence they encode, often resulting in greater structural stability (Yang and Zhang 2015). Therefore, lncRNAs frequently contain multiple structural and sequence elements that modulate their molecular interactions (Rivas et al. 2016). These structural motifs may be evolutionarily conserved as long as the complementary base pairs that form them remain unchanged (sequence covariation) (Ramírez‐Colmenero et al. 2020).

TERC contains a helix subdomain embedded within a pseudoknot structure, conserved from Japanese rice fish to humans due to its critical interaction with the telomerase enzyme (Wang et al. 2016). Libra is a brain‐expressed transcript that originated in the basal vertebrate lineage and retains a recognition site that regulates miR‐29 degradation. Knockdown of Libra results in the accumulation of miR‐29 during cerebellar development, impairing both motor function and learning (Bitetti et al. 2018).

Computational analyses have identified conserved elements in several lncRNAs—for example, Cyrano and CHASERR show conserved regions across vertebrates (Ross et al. 2021), and multiple motifs have been predicted in MALAT1 using in silico approaches (McCown et al. 2019). Predictions based solely on sequence or motif conservation remain controversial, structural studies of the well‐characterized lncRNA HOTAIR, found insufficient evidence of covariation to support structural conservation (Rivas et al. 2016). Similarly, although NEAT1 has a conserved role in paraspeckle scaffolding, motif analyses revealed only small microdomains of structural conservation, which appear insufficient to fully explain its preserved function (Lin et al. 2018). Still, some researchers suggest that broader lineage sampling and improved computational methods could enhance the power of sequence conservation analyses (Tavares et al. 2019).

## 7 Challenges and Opportunities for Studying lncRNAs in Non‐Model Species

LncRNAs are key regulators of gene expression during development. HTS allows identification of novel lncRNAs across diverse species, yet this progress is hindered by the lack of high‐quality reference genomes and transcriptomes in non‐model organisms. Generating well‐assembled genomes and transcriptomic data across tissues, developmental stages, and sexes is essential for characterizing transcripts by their genomic position, exon‐intron structure, and coding potential.

The heterogeneity of lncRNAs (genomic position, subcellular localization, inconsistent definitions and complex conservation) has led to ambiguities in nomenclature (Mattick et al. 2023), complicating cross‐species comparisons. Although curated databases now assign standardized gene IDs, comprehensive resources remain scarce for many vertebrates beyond traditional models like zebrafish, Xenopus, chicken, and mouse. Broader adoption of these repositories will support the creation of robust comparative frameworks akin to those used for PCGs.

Identifying novel lncRNAs remains difficult due to their low expression, poor conservation, and limited annotation. Most pipelines remove transcripts overlapping protein‐coding regions or use coding potential predictors typically trained on model species, posing challenges when applied to non‐model organisms. Benchmarking these tools and integrating structural domain predictions can improve lncRNA candidate identification.

Experimental validation of lncRNAs (e.g., knockouts, overexpression) is often prohibitive due to cost and scalability. Therefore, differential expression analyses help infer function by linking lncRNAs to specific phenotypes or better‐annotated molecules like mRNAs or miRNAs. To study lncRNA function, deep‐learning tools can predict secondary structures or molecular interactions without requiring prior annotations. However, these tools may overfit training sets, underscoring the need for diverse experimental data to increase performance across lineages.

Evolutionary conservation studies starting with sequence similarity in closely related species should expand to include secondary structure and positional conservation in more distantly related taxa (Tavares et al. 2019). This further highlight the need for high‐quality reference annotations across phylogenies. As computational tools improve and sampling broadens, especially among vertebrates, major advances in lncRNA biology are anticipated.

### 7.1 Recommended Workflow

**FIGURE 3:**
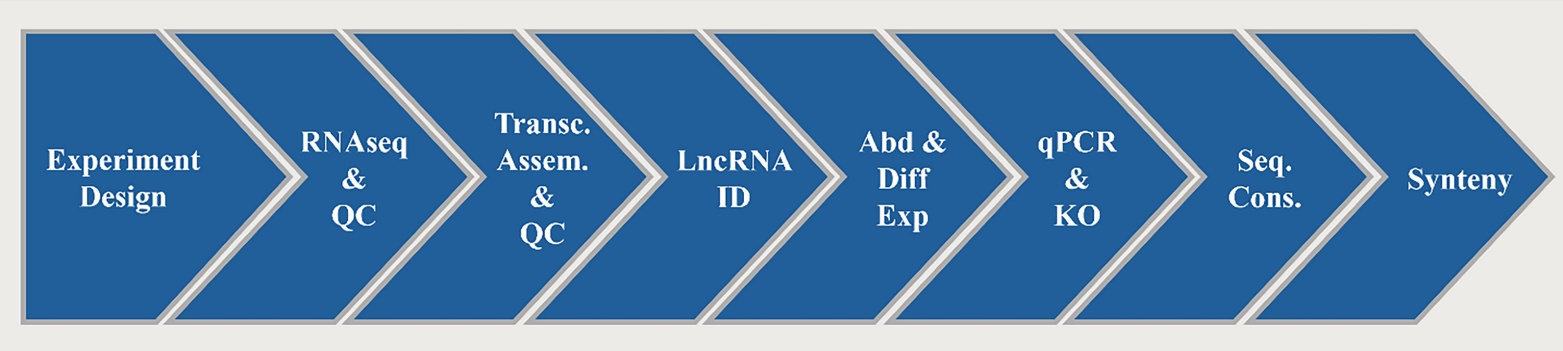
Suggested workflow for a lncRNA identification and analysis. When working with lncRNAs, experimental design is the critical first step, as it determines all downstream analyses. An appropriate RNA sequencing strategy must be selected to adequately capture the biological complexity of lncRNAs. Transcriptome assembly should be performed considering isoform complexity, available computational resources, and whether a reference genome is available. LncRNAs can then be identified using the tools and filtering criteria described above. Abundance estimation and differential expression analyses may reveal transcriptional patterns associated with the biological phenomenon under study. Candidate lncRNAs can be further validated using quantitative PCR and functional assays such as knockdown or knockout approaches. Finally, evolutionary conservation can be assessed. A practical starting point is evaluating sequence conservation across short phylogenetic distances and when genomic resources are available, synteny analyses can provide stronger evidence for lncRNA conservation, particularly in cases where primary sequence conservation is limited.

Due to the biological complexity and relative novelty of studying lncRNAs, we propose a simplified workflow that can serve as a flexible template and be adapted according to the specific research question (Figure 3).

#### 7.1.1 Experimental Design

Depending on the biological question, researchers may either pool multiple tissues to maximize RNA yield and transcript diversity or analyze individual tissues to obtain a more precise view of tissue‐specific expression patterns. The latter approach may be particularly relevant for lncRNAs, which often exhibit high spatiotemporal specificity (Mattick et al. 2023). An important subsequent step is determining an appropriate sample size. As a general guideline for non‐model species—where biological material may be limited—a minimum of three biological replicates per comparison group is recommended. When feasible, six replicates provide improved robustness, and statistical power increases substantially with larger sample sizes (e.g., 12 samples per group) (Schurch et al. 2016).

#### 7.1.2 RNA‐Sequencing

When using RNA‐seq for lncRNA identification, several technical considerations are essential. Because many lncRNAs lack polyadenylation, total RNA sequencing coupled with rRNA depletion is recommended over poly(A)‐selection, as it can recover approximately twice the number of transcripts compared to poly(A)‐enriched protocols (Guo et al. 2015). Furthermore, since numerous lncRNAs are transcribed antisense to PCGs, strand‐specific (directional) libraries are strongly recommended to accurately distinguish overlapping transcripts (Mills et al. 2013). In non‐model species lacking high‐quality reference genomes, combining short‐ and long‐read sequencing technologies is advantageous. Long reads facilitate the resolution of repetitive or structurally complex regions and improve transcript structure annotation, whereas short reads provide higher sequencing depth and more accurate transcript quantification (Kainth et al. 2023).

#### 7.1.3 Transcriptome Assembly

Currently, there are multiple options for both reference‐guided and de novo transcriptome assembly. Among the most widely used tools when a reference genome is available is StringTie2, which has demonstrated strong performance handling both short‐ and long‐read sequencing technologies (Kovaka et al. 2019) Similarly, numerous reference‐free assemblers are available, differing in speed, scalability, memory requirements, and computational demands. These tools also vary in how they manage highly repetitive sequences and complex isoform structures—two particularly important challenges in transcriptome reconstruction. Given these differences, we recommend consulting the comprehensive benchmarking study by Hölzer and Marz (2019) which systematically compares transcriptome assemblers and provides practical guidance for selecting the most appropriate tool based on experimental design and available computational resources.

#### 7.1.4 Quality Control

After transcriptome assembly, the next steps typically include quality control, alignment and abundance estimation, and alignment thinning. Quality control involves evaluating statistics that reflect assembly continuity and biological completeness. For example, EX90N50—described in Hölzer and Marz (2019)—assesses contiguity while accounting for transcript expression levels. In addition, representation of annotated genes can be evaluated using conserved single‐copy orthologues with BUSCO (Manni et al. 2021), which provides an estimate of transcriptome completeness based on evolutionarily conserved high‐confidence annotated gene sets.

#### 7.1.5 LncRNA Identification

Following quality assessment, we recommend proceeding with lncRNA identification. As mentioned above, a robust strategy is to combine at least two complementary prediction tools to maximize the detection of potential novel lncRNAs while minimizing false positives. Authors are encouraged to consult benchmarking studies such as Singh and Roy (2022) and Zheng et al. (2021) when selecting appropriate tools. These studies systematically compare commonly used predictors and highlight trade‐offs in sensitivity, specificity, computational requirements, and ease of implementation. Tool selection should consider computational expertise, hardware availability, transcriptome completeness, and the biological system under study.

Similarly, reads or assembled transcripts can be aligned back to a reference genome, transcriptome, or curated database to identify known coding and non‐coding transcripts and infer their potential roles. This annotation step helps distinguish previously described lncRNAs from putative novel candidates and reduces redundancy prior to downstream expression, coexpression, or functional analyses.

#### 7.1.6 Abundance Estimation and Expression Analyses

After transcript annotation, transcript abundance can be estimated prior to downstream differential expression analysis, as explained above. A comprehensive overview of these processes—including commonly used tools, methodological considerations, normalization strategies, and practical recommendations—is provided by Raghavan et al. (2022) who also offer additional resources for deeper exploration of RNA‐seq analytical workflows across diverse biological systems.

Differential expression analysis can subsequently be integrated with pairwise correlation analyses to infer potential direct regulatory interactions under the “guilt‐by‐association” framework. In this context, transcripts displaying highly correlated expression patterns across biological conditions may participate in shared regulatory programs or functional pathways.

Likewise, Weighted Gene Co‐expression Network Analysis can be implemented to identify broader co‐expression modules rather than isolated transcript pairs. These modules group genes according to shared expression dynamics and can then be subjected to functional enrichment analyses to uncover biologically relevant processes or pathways potentially associated with candidate lncRNAs. For an accessible and thorough discussion of gene co‐expression analysis principles, assumptions, limitations, and best‐practice guidelines, we recommend the work by Zogopoulos et al. (2022).

#### 7.1.7 Experimental Validation

Expression validation should first confirm transcript presence and differential abundance. Quantitative reverse transcription PCR (qRT‐PCR) remains one of the most accessible and widely applicable approaches for this purpose. However, particular care must be taken when designing primers for lncRNAs, as they often exhibit low expression levels, tissue specificity, and potential isoform complexity. For detailed methodological considerations see Kolenda et al. (2019).

Expression validation alone does not demonstrate function. Therefore, complementary loss‐of‐function approaches such as siRNA‐, shRNA‐, or antisense oligonucleotide‐mediated knockdown can provide further support for regulatory roles. These strategies allow researchers to evaluate downstream transcriptional or phenotypic effects following lncRNA depletion. A recent overview of experimental approaches and considerations for lncRNA functional interrogation is provided by Khaleel et al. (2025).

#### 7.1.8 Evolutionary Conservation Analyses

Due to the generally low primary sequence conservation of lncRNAs across large evolutionary distances, conservation analyses should begin at relatively shallow phylogenetic scales. A practical first step is to perform BLAST searches against genomes or transcriptomes from closely related species to identify putative homologous loci.

Once candidate homologues are identified, a structure‐aware multiple sequence alignment approach is recommended. Tools such as LocARNA (Will 2024) allow the simultaneous alignment of RNA sequences while incorporating predicted secondary structure information. This is particularly relevant for lncRNAs, whose functional conservation may reside more in structural motifs than in strict sequence identity. Such alignments can reveal conserved stem–loop structures, short structural domains, or compensatory mutations that preserve base pairing despite nucleotide substitutions.

The resulting multiple alignment can then serve as input for covariance model construction using the Infernal toolkit (Nawrocki and Eddy 2013). Covariance models integrate both sequence and secondary structure information to identify conserved RNA families and generate consensus structural profiles. These models can subsequently be used to scan additional genomes for more distantly related homologues, thereby strengthening evolutionary inferences.

Alternatively, when extensive and well‐curated genomic annotations are available, a syntenic conservation analysis can provide additional support for the evolutionary conservation of lncRNAs (Camilleri‐Robles et al. 2022). To do so, genomic annotation must be of high quality for the location of the lncRNA and its flanking PCGs. Once these elements are clearly defined, the next step is to identify orthologues of the flanking PCGs in other species.

After orthology relationships are established, one can then evaluate whether a non‐coding transcript is present in the corresponding species within the same syntenic interval and whether it is transcribed from the same strand. The consistent production of a transcript from the same syntenic neighborhood across different species may indicate that the lncRNA is evolutionarily conserved, even in cases where little or no primary sequence conservation is detectable. This positional stability suggests functional constraint acting at the genomic context level rather than at the nucleotide level (Nitsche and Stadler 2017; Ranjan et al. 2025). For methodological approaches and practical examples of this framework applied to lncRNA evolutionary studies, see Bush et al. (2018) and Olazagoitia‐Garmendia et al. (2023).

## 8 Conclusion

LncRNAs play central, yet poorly understood, roles in orchestrating complex developmental transitions like metamorphosis. In non‐model vertebrates, these processes provide a unique window into the regulatory versatility and evolutionary innovation of lncRNAs, whose full biological significance is only beginning to emerge. Advancing lncRNA research demands integrative approaches that combine computational prediction, evolutionary analysis, and experimental validation. To overcome challenges such as low conservation rates, incomplete annotations, and algorithmic biases researchers must prioritize broader taxonomic and developmental sampling, especially in non‐model organisms, alongside robust functional assays.

## Funding

This work was supported by Universidad Nacional Autónoma de México, IN219925 and Secretaría Nacional de Ciencia, Tecnología e Innovación, 1096935.

## Data Availability

Data sharing not applicable to this article as no datasets were generated or analyzed during the current study.
